# Supplementary material for: Immune Responses to SARS-CoV-2 Variants WT and XBB.1.9: Assessing Vulnerabilities and Preparedness
Source: Vaccines (Basel). 2025 Nov 16;13(11):1167. doi: 10.3390/vaccines13111167 (PMC12656813; doi:10.3390/vaccines13111167)
Supplement: Supplementary file 1 [file vaccines-13-01167-s001.zip › vaccines-3927927-supplementary.pdf]

Supplementary Table S1. Summary of nucleotide differences between the SARS-CoV-2 XBB lineage and the Wuhan reference genome (NC_045512.2). The table lists all lineage-defining substitutions and deletions across the viral genome. Variant positions were determined based on a consensus of sequences designated as XBB in GISAID and compared to the Wuhan reference sequence.

Refference	Position	Mutation	Genetic_region	Mutation_type	AA_sub
C	241	T	UTR5	non coding SNP	
A	405	G	NSP1	nonsynonymous	K47R
G	510	A	NSP1	nonsynonymous	G82D
T	670	G	NSP1	nonsynonymous	S135R
C	2790	T	NSP3	nonsynonymous	T24I
C	3037	T	NSP3	synonymous	F106F
G	4184	A	NSP3	nonsynonymous	G489S
C	4321	T	NSP3	synonymous	A534A
C	9344	T	NSP4	nonsynonymous	L264F
A	9424	G	NSP4	synonymous	V290V
C	9534	T	NSP4	nonsynonymous	T327I
C	9866	T	NSP4	nonsynonymous	L438F
C	10029	T	NSP4	nonsynonymous	T492I
C	10198	T	NSP5	synonymous	D48D
G	10447	A	NSP5	synonymous	R131R
C	10449	A	NSP5	nonsynonymous	P132H
T	11288	-	NSP6	deletion	SGF106-
C	11289	-	NSP6	deletion	SGF106-
T	11290	-	NSP6	deletion	SGF106-
G	11291	-	NSP6	deletion	SGF107-
G	11292	-	NSP6	deletion	SGF107-
T	11293	-	NSP6	deletion	SGF107-
T	11294	-	NSP6	deletion	SGF108-
T	11295	-	NSP6	deletion	SGF108-
T	11296	-	NSP6	deletion	SGF108-
C	12880	T	NSP9	synonymous	I65I
C	14408	T	NSP12b	nonsynonymous	P314L
G	15451	A	NSP12b	nonsynonymous	G662S
C	15714	T	NSP12b	synonymous	L749L
C	15738	T	NSP12b	synonymous	F757F
T	15939	C	NSP12b	synonymous	D824D
T	16342	C	NSP13	nonsynonymous	S36P
C	17410	T	NSP13	nonsynonymous	R392C
T	17859	C	NSP13	synonymous	Y541Y
A	18163	G	NSP14	nonsynonymous	I42V
A	19326	G	NSP14	synonymous	P429P

C	19955	T	NSP15	nonsynonymous	T112I
A	20055	G	NSP15	synonymous	E145E
C	21618	T	SPIKE	nonsynonymous	T19I
T	21633	-	SPIKE	deletion	LPPA24S
A	21634	-	SPIKE	deletion	LPPA24S
C	21635	-	SPIKE	deletion	LPPA25S
C	21636	-	SPIKE	deletion	LPPA25S
C	21637	-	SPIKE	deletion	LPPA25S
C	21638	-	SPIKE	deletion	LPPA26S
C	21639	-	SPIKE	deletion	LPPA26S
T	21640	-	SPIKE	deletion	LPPA26S
G	21641	-	SPIKE	deletion	LPPA27S
T	21810	C	SPIKE	nonsynonymous	V83A
G	21987	A	SPIKE	nonsynonymous	G142D
T	21992	-	SPIKE	deletion	Y144-
A	21993	-	SPIKE	deletion	Y144-
T	21994	-	SPIKE	deletion	Y144-
C	22000	A	SPIKE	nonsynonymous	H146Q
C	22109	G	SPIKE	nonsynonymous	Q183E
T	22200	A	SPIKE	nonsynonymous	V213E
G	22577	C	SPIKE	nonsynonymous	G339H
G	22578	A	SPIKE	nonsynonymous	G339H
G	22599	C	SPIKE	nonsynonymous	R346T
C	22664	A	SPIKE	nonsynonymous	L368I
C	22674	T	SPIKE	nonsynonymous	S371F
T	22679	C	SPIKE	nonsynonymous	S373P
C	22686	T	SPIKE	nonsynonymous	S375F
A	22688	G	SPIKE	nonsynonymous	T376A
G	22775	A	SPIKE	nonsynonymous	D405N
A	22786	C	SPIKE	nonsynonymous	R408S
G	22813	T	SPIKE	nonsynonymous	K417N
T	22882	G	SPIKE	nonsynonymous	N440K
G	22895	C	SPIKE	nonsynonymous	V445P
T	22896	C	SPIKE	nonsynonymous	V445P
G	22898	A	SPIKE	nonsynonymous	G446S
T	22942	G	SPIKE	nonsynonymous	N460K
G	22992	A	SPIKE	nonsynonymous	S477N
C	22995	A	SPIKE	nonsynonymous	T478K
A	23013	C	SPIKE	nonsynonymous	E484A
T	23019	C	SPIKE	nonsynonymous	F486S
T	23031	C	SPIKE	nonsynonymous	F490S
A	23055	G	SPIKE	nonsynonymous	Q498R

A	23063	T	SPIKE	nonsynonymous	N501Y
T	23075	C	SPIKE	nonsynonymous	Y505H
A	23403	G	SPIKE	nonsynonymous	D614G
C	23525	T	SPIKE	nonsynonymous	H655Y
T	23599	G	SPIKE	nonsynonymous	N679K
C	23604	A	SPIKE	nonsynonymous	P681H
C	23854	A	SPIKE	nonsynonymous	N764K
G	23948	T	SPIKE	nonsynonymous	D796Y
A	24424	T	SPIKE	nonsynonymous	Q954H
T	24469	A	SPIKE	nonsynonymous	N969K
C	25000	T	SPIKE	synonymous	D1146D
C	25416	T	ORF3a	synonymous	F8F
C	25584	T	ORF3a	synonymous	T64T
C	26060	T	ORF3a	nonsynonymous	T223I
C	26270	T	ENVELOPE	nonsynonymous	T9I
A	26275	G	ENVELOPE	nonsynonymous	T11A
C	26577	G	MEMBRANE	nonsynonymous	Q19E
G	26709	A	MEMBRANE	nonsynonymous	A63T
C	26858	T	MEMBRANE	synonymous	F112F
A	27259	C	ORF6	synonymous	R20R
G	27382	C	ORF6	nonsynonymous	D61L
A	27383	T	ORF6	nonsynonymous	D61L
T	27384	C	ORF6	nonsynonymous	D61L
C	27807	T	ORF7b	synonymous	C138C
A	28271	T	extragenic	non coding SNP	
C	28311	T	NUCAP	nonsynonymous	P13L
G	28362	-	NUCAP	deletion	GERS30G
A	28363	-	NUCAP	deletion	GERS30G
G	28364	-	NUCAP	deletion	GERS31G
A	28365	-	NUCAP	deletion	GERS31G
A	28366	-	NUCAP	deletion	GERS31G
C	28367	-	NUCAP	deletion	GERS32G
G	28368	-	NUCAP	deletion	GERS32G
C	28369	-	NUCAP	deletion	GERS32G
A	28370	-	NUCAP	deletion	GERS33G
G	28881	A	NUCAP	nonsynonymous	R203K
G	28882	A	NUCAP	nonsynonymous	R203K
G	28883	C	NUCAP	nonsynonymous	G204R
A	29510	C	NUCAP	nonsynonymous	S413R
